# 60558 Non-Suicidal Self Injury in Military Veterans with PTSD: An Ecological Momentary Assessment Study

**DOI:** 10.1017/cts.2021.757

**Published:** 2021-03-30

**Authors:** Lorig Kachadourian, Tami Sullivan, Robert Pietrzak

**Affiliations:** 1 VA Connecticut Healthcare System and Yale University; 2 Yale University

## Abstract

ABSTRACT IMPACT: This study will help determine whether ecological momentary assessment is feasible in assessing changes in negative affect and the occurrence of non-suicidal self-injury (NSSI) in military Veterans with post-traumatic stress disorder; if so it will allow for further examination of correlates of NSSI which will inform treatment efforts. OBJECTIVES/GOALS: Given the advantages of ecological momentary assessment (EMA), and the lack of research on non-suicidal self-injury (NSSI) in military populations, the goal of the current pilot study is to determine the feasibility of using EMA to assess daily changes in post-traumatic stress disorder (PTSD) symptoms, negative affect and NSSI in veterans with PTSD. METHODS/STUDY POPULATION: Twenty military veterans with post-traumatic stress disorder (PTSD) who have engaged in non-suicidal self-injury (NSSI) in the previous 12 months will be recruited. Participants will complete assessments 4 times per day for 28 days at randomly scheduled times. Assessments will measure PTSD symptoms, negative emotions, and NSSI urges and behaviors. At the conclusion of the 28-day study period, participants will complete measures that ask about their experiences in the study, (e.g., the acceptability of the daily surveys and the accessibility of the web-based surveys). Feasibility will be determined with regard to the success of recruiting eligible participants and compliance with daily survey completion. Variability in PTSD symptoms, negative affect, and NSSI urges and behaviors also will be determined. RESULTS/ANTICIPATED RESULTS: It is anticipated that this study will successfully recruit 20 veterans with PTSD with a history of engaging in NSSI within the previous 12 months. It is also anticipated that daily survey completion rates will be approximately 90% based on previous research using EMA with veterans with PTSD and that participants will indicate satisfaction with the procedures of the study. It is anticipated that participants will demonstrate variability in PTSD symptoms, indicated by changes in the number of symptoms endorsed and the intensity of those symptoms experienced. Finally, it is anticipated that participants will demonstrate variability in negative emotions (fear, hostility, guilt, and sadness).

DISCUSSION/SIGNIFICANCE OF FINDINGS: Findings from this study will support the use of EMA in a subsequent large-scale investigation examining time-varying symptoms of PTSD and negative affect as antecedents to NSSI. Information from this large-scale study will in turn be used to inform treatments that may to decrease NSSI in veterans by targeting specific symptoms and negative emotions.

